# Manipulation of starch bioaccessibility in wheat endosperm to regulate starch digestion, postprandial glycemia, insulinemia, and gut hormone responses: a randomized controlled trial in healthy ileostomy participants^1^,^2^

**DOI:** 10.3945/ajcn.114.106203

**Published:** 2015-09-02

**Authors:** Cathrina H Edwards, Myriam ML Grundy, Terri Grassby, Dafni Vasilopoulou, Gary S Frost, Peter J Butterworth, Sarah EE Berry, Jeremy Sanderson, Peter R Ellis

**Affiliations:** 3 Biopolymers Group, Diabetes and Nutritional Sciences Division, King’s College London, London, United Kingdom;; 4 Department of Gastroenterology, Guy's and St. Thomas’ National Health Service Foundation Trust, London, United Kingdom; and; 5 Nutrition and Dietetic Research Group, Faculty of Medicine, Hammersmith Campus, Imperial College, London, United Kingdom

**Keywords:** ileostomy, starch, postprandial, glycemia, bioaccessibility, structure, digestion

## Abstract

**Background:** Cereal crops, particularly wheat, are a major dietary source of starch, and the bioaccessibility of starch has implications for postprandial glycemia. The structure and properties of plant foods have been identified as critical factors in influencing nutrient bioaccessibility; however, the physical and biochemical disassembly of cereal food during digestion has not been widely studied.

**Objectives:** The aims of this study were to compare the effects of 2 porridge meals prepared from wheat endosperm with different degrees of starch bioaccessibility on postprandial metabolism (e.g., glycemia) and to gain insight into the structural and biochemical breakdown of the test meals during gastroileal transit.

**Design:** A randomized crossover trial in 9 healthy ileostomy participants was designed to compare the effects of 55 g starch, provided as coarse (2-mm particles) or smooth (<0.2-mm particles) wheat porridge, on postprandial changes in blood glucose, insulin, C-peptide, lipids, and gut hormones and on the resistant starch (RS) content of ileal effluent. Undigested food in the ileal output was examined microscopically to identify cell walls and encapsulated starch.

**Results:** Blood glucose, insulin, C-peptide, and glucose-dependent insulinotropic polypeptide concentrations were significantly lower (i.e., 33%, 43%, 40%, and 50% lower 120-min incremental AUC, respectively) after consumption of the coarse porridge than after the smooth porridge (*P* < 0.01). In vitro, starch digestion was slower in the coarse porridge than in the smooth porridge (33% less starch digested at 90 min, *P* < 0.05, paired *t* test). In vivo, the structural integrity of coarse particles (∼2 mm) of wheat endosperm was retained during gastroileal transit. Microscopic examination revealed a progressive loss of starch from the periphery toward the particle core. The structure of the test meal had no effect on the amount or pattern of RS output.

**Conclusion:** The structural integrity of wheat endosperm is largely retained during gastroileal digestion and has a primary role in influencing the rate of starch amylolysis and, consequently, postprandial metabolism. This trial was registered at isrctn.org as ISRCTN40517475.

## INTRODUCTION

Diets containing a high proportion of foods with a low glycemic index (GI)^7^ are associated with reduced cardiometabolic risk factors (1, 2) and may also encourage weight loss and support obesity management (3). Wheat is the main ingredient in a range of staple foods that form part of a healthy diet (4); however, many wheat-based staples (e.g., certain breads and breakfast cereals) have a high GI (5). Differences in glycemic responses to starch-rich foods such as wheat products depend highly on the rate and extent to which available starch is digested by α-amylase and made available for absorption in the small intestine (6–8). Understanding the mechanisms of digestion and absorption in the small intestine may therefore suggest ways of manipulating existing foods and/or ingredients to control metabolic responses.

Compelling evidence reveals that food structure plays a critical role in influencing the bioaccessibility of starch and other macronutrients and the subsequent metabolic responses (9–11). Currently, however, there is a lack of understanding of how foods are structurally disassembled during digestive transit. Many foods are consumed as plant tissues, consisting of nutrients enclosed within plant cells. Plant cell walls (dietary fiber) are resistant to digestion in the upper gastrointestinal tract and may, if structurally intact, protect the enclosed nutrients from digestive enzymes, thereby delaying or limiting bioaccessibility (12, 13). Indeed, intact plant cells still containing nutrients (e.g., starch, lipid, β-carotene) have been identified in ileal effluent after plant foods were consumed (10, 12, 14, 15). Previous in vivo studies have also reported attenuated glycemia and insulinemia in healthy participants after ingestion of structurally intact foods compared with a destructured equivalent (e.g., mainly ruptured cells) (9, 16, 17).

A common misconception is that any food containing whole grains, even in flour form, is slowly digested. However, the high GI of white and whole-meal wheat bread is linked to structural characteristics of the constituent flour, which contains mostly ruptured cells with high-bioaccessibility starch (18, 19). Previous studies suggest that inclusion of high proportions of intact whole grains or soluble dietary fiber in bread may attenuate glycemic responses (8). However, to what extent starch bioaccessibility and glycemia can be controlled by manipulating the structural integrity of wheat endosperm, the most commonly consumed dietary source of starch, is not yet known.

The aim of this postprandial ileostomy study was to provide insight into the structural and biochemical degradation of wheat endosperm (i.e., the starch storage tissue of wheat grain) during gastroileal digestion and its effects on metabolic responses (e.g., glycemia, insulinemia, and gut hormone release). We hypothesized that porridge prepared from coarsely milled wheat endosperm (containing intact cells) would be digested more slowly and therefore elicit a lower glycemic response than a nutritionally matched porridge prepared from finely milled endosperm (lacking intact cells). This unique study in healthy participants with an ileostomy has provided new insight into both the mechanistic aspects of starch digestion and its metabolic consequences.

## METHODS

### Participants

Healthy participants aged 20–76 with ileostomies were recruited between September 2012 and July 2013 via the Ileostomy Association in London. All participants had undergone a proctocolectomy for ulcerative colitis, pure colonic Crohn disease, or lower bowel cancer at least 1 y before taking part in the study. The study protocol was registered as ISRCTN40517475 and approved by the relevant ethics committee in the United Kingdom (REC 12/LO/1016). All volunteers gave their written informed consent after being provided with oral and written information about the aims and protocol of the study.

All participants had healthy small bowels with well-functioning ileostomies with <10 cm of small intestine resected. Exclusion criteria included the following: BMI (in kg/m^2^) <20 or >35, fasting plasma glucose >7 mmol/L, serum cholesterol >7.8 mmol/L, or serum triacylglycerols >3 mmol/L. Fasting plasma glucose concentration, BMI, blood pressure, liver function, and blood cell counts were confirmed to be within prescribed limits before enrollment in the study. All participants were otherwise healthy, with no history of diabetes or signs of diagnosed gastrointestinal conditions, as confirmed by means of a full medical history and examination. The usual dietary habits of individuals were assessed from a 3-d diet diary, and the nutrient intake was analyzed with the use of NetWisp v.3.0 (Tinuviel Software).

This study was one part of a 2-branch investigation (study 1 was on lipid bioaccessibility and study 2 on starch bioaccessibility) with common participant recruitment and screening processes for both branches. The participant characteristics, study details, and results presented in this publication are for the starch study only.

### Study design

A randomized crossover study design was used in which each participant received 2 experimental meals (in random sequence) separated by at least 1 wk. The random allocation of the treatment sequence was based on a computer-generated random number sequence. The study investigators generated the random allocation sequence, enrolled participants, and assigned participants to interventions. Because of the different physical appearance and texture of the test meals, study participants were not blinded; however, all samples were coded so that those investigators assessing outcomes (e.g., performing sample and data analysis) were blinded. All screening and study visits took place at the Clinical Research Facilities at St. Thomas’ Hospital, London.

On the day preceding the study, participants were provided with a standardized low-fiber evening meal and advised to drink plenty of water. Participants were also instructed to avoid food and drink high in sugar, caffeine, or alcohol and to refrain from strenuous exercise both on the day preceding the study and throughout the study period.

**FIGURE 1:**
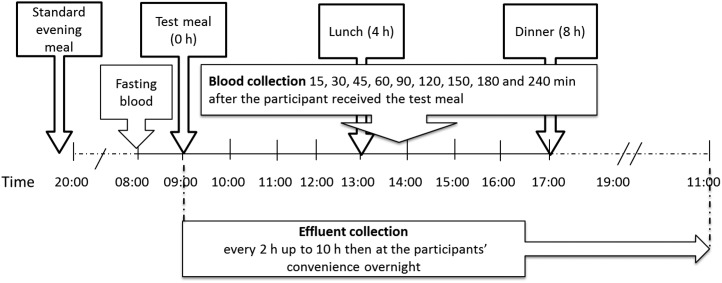
Blood and effluent collection time points and meal times. Fasting blood samples for baseline measurements were collected at −15 min (glucose only) and, for all analytes, immediately before the test meal (0 min). Blood samples thereafter were collected at regular intervals up to 4 h, and ileal effluent was collected every 2 h up to 10 h. Overnight effluent samples (between 2200 and 1100 the next day) were collected at variable time points, at the participants’ convenience. Effluent samples were preserved in ethanol or by freezing at the time of collection to inhibit enzymatic and chemical deterioration of carbohydrates (e.g., starch).

An overview of the study day protocol is provided in **Figure 1**. On the day of the study visit, participants arrived at the clinical research facilities after a 12-h fast. A cannula was inserted into a forearm vein, and a fasted venous blood glucose sample (−15 min) was obtained. Participants were then required to either completely empty their ileostomy pouch or attach a new pouch. Additional fasted venous blood samples (time = 0 min) were then obtained for glucose, insulin, C-peptide, triacylglycerols, and nonesterified fatty acids (NEFAs), as well as gut hormone analyses, after which the test meal was consumed within 15 min. Blood samples thereafter were taken at 15, 30, 45, 60, 90, 120, 150, 180, and 240 min. Ileal effluent was collected at 2, 4, 8, and 10 h, and overnight.

Meals low in resistant starch (RS) were provided after blood and effluent collections at 4 h, and again at 8 h (dinner with dessert), to minimize interference with ileal RS measurements. Participants were required to eat an identical menu for both study visits. Foods for all meals were purchased from Sainsbury’s, London. Lunch (621 kcal) consisted of a leafy salad with cheese and either tofu, chicken breast, beef, or tuna, served with yogurt and juice (from concentrate), and contained <4 g starch. Dinner consisted of mashed potato with fish, chicken, lamb, or beef and cooked carrots, and was followed by a high-calorie (360–420-kcal/portion) dessert. Dinner with dessert contained up to 50 g starch in total, but the majority (at least 85%) of the starch in this meal originated from mashed potato, which contained starch that previously was found to be readily digested and absorbed (20).

For the a priori power calculations, we assumed a difference between mean glucose incremental AUC of at least 20% and an SD of the difference of 18%. These values were based on data from previous studies, as well as the observed differences in in vitro digestibility (i.e., a predictor of glycemia) of the specific test meals (6). This calculation indicated that, with a repeated-measures study design, 9 subjects would give 90% power at an α-level of 0.05. Changes in plasma glucose concentrations and serum insulin and C-peptide responses were regarded as primary outcomes, and lipemic responses (triacylglycerols and NEFAs) and RS contents of ileal effluent were secondary outcomes. Changes in gut hormone concentrations, specifically plasma glucose-dependent insulinotropic polypeptide (GIP), polypeptide YY (PYY), glucagon-like peptide 1 (GLP-1), and cholecystokinin, were regarded as exploratory outcomes.

### Formulation and preparation of test meals

Test meals consisted of 2 freshly prepared porridges made from 77 g durum wheat endosperm, cooked in water and flavored with 17 g low-sugar black currant jam (Sainsbury’s Reduced Sugar Blackcurrant Jam) and 61 g jelly (Hartley’s Low Calorie Ready To Eat Blueberry and Blackcurrant Jelly). Proximate analysis of the durum wheat (protein, fat, dietary fiber by the Association of Official Analytical Chemists method, ash, and moisture) was performed by Premier Analytic Services, and additional direct measurements of available carbohydrate (starch and sugar) contents were performed with the use of a modified Megazyme kit (Megazyme International) method (18). On the basis of data from proximate analysis of wheat and the nutrition declaration on food packaging (for the jam and jelly), each porridge meal (155 g solids with 449 mL water) provided a total of 57.8 g available carbohydrate (composed of 55.4 g starch and 2.4 g sugar), 5.2 g dietary fiber, 9.3 g protein, 1.6 g fat, and 0.2 g salt.

The durum wheat endosperm component of the meal was provided in the form of finely milled flour (<0.2 mm) to create a smooth porridge or coarsely milled macroparticles (∼2 mm) to create a coarse porridge. The smooth and coarse porridges were predicted to be high- and low-bioaccessibility starch test meals, respectively. The endosperm material was prepared from de-branned durum wheat grains with the use of a Satake TH050 roller-mill (Satake Europe), as described previously (18). Microstructural analysis (**Supplemental Figure 1**) confirmed that the coarsely milled particles contained a high proportion of intact cells, whereas particles <0.21 mm consisted almost entirely of ruptured cells. Durum wheat (*Triticum durum* L. “Svevo”), donated by Millbo, was selected for this study because its fracture properties make it particularly well-suited to the milling techniques used.

The porridges were cooked under standardized heating conditions by simmering smooth or coarse wheat endosperm in water (while stirring vigorously) to achieve total and uniform gelatinization of the starch. Next, the porridge was allowed to cool for 5 min, during which time the jam and jelly flavoring was stirred in. The liquid component of the meal was standardized at 449 mL by providing the required volume of drinking water to make up for evaporative losses. The meal was served immediately thereafter to prevent further starch retrogradation, which is known to decrease the susceptibility of starch to amylolysis (21). Both test meals were designed to be of a particle size and consistency that could comfortably be swallowed whole without prior mastication. Participants were instructed to swallow the porridge with minimal chewing and were required to consume the entire meal within 15 min of serving.

### In vitro starch digestibility of test meals

The rate at which starch amylolysis products become bioaccessible (i.e., available for absorption) during duodenal digestion is an important determinant of the duration and magnitude of the glycemic response (6, 7, 22, 23). In particular, the extent of starch digested at 90 min has previously been reported to correlate well with the glucose response (AUCs for 120 min) (24). Because of the difficulty in studying luminal digestion of starch in vivo, the rate and extent of starch amylolysis of the 2 test meals was determined in vitro with the use of our well-established digestion protocol (18), which involves sample digestion with porcine pancreatic α-amylase and quantification of digestion products with the use of the Prussian blue method (25). Logarithm of slope analysis (18, 26) was applied to the digestibility data and used to predict the total extent of digestion (*C*_∞_). Because the Prussian blue assay quantifies products as maltose (not glucose) and either underestimates or fails to account for other minor starch digestion products (maltotriose and α-limit dextrins), it slightly underestimates the extent of overall starch digestion. For purposes of comparison, therefore, all digestibility data were expressed as a percentage of total hydrolyzable starch based on maltose equivalents, with the use of extracted durum wheat starch, which had been hydrothermally processed to fully gelatinize the starch (18), as a reference material. Thus, the starch reference sample represented 100% hydrolysis. The proportion of hydrolyzable starch digested after 90 min, known as the hydrolysis index (HI), was estimated from the first-order kinetic model describing the in vitro data (18).

### Collection and handling of blood samples

Venous blood samples for glucose, insulin, C-peptide, triacylglycerols, and NEFA analyses were collected and handled as described previously (27). Blood samples for gut hormone analyses were dispensed into K_2_ EDTA-coated tubes preloaded with either 100 μL aprotinin (10,000 KIU/mL; Nordic Pharma) for GIP and PYY analyses or 40 μL dipeptidyl-peptidase-IV inhibitor (Merck Millipore) for GLP-1 and cholecystokinin analyses, and kept on ice for 15 min followed by centrifugation and plasma storage. All blood samples were centrifuged at 1500 × *g* for 15 min at 4°C, and aliquots of the supernatant were collected and stored at either −40°C (for glucose, insulin, C-peptide, triacylglycerols, and NEFAs) or −80°C (for gut hormones) until analyzed.

### Collection and handling of effluent samples

Ileal effluent was collected every 2 h for up to 10 h and overnight at the convenience of participants. Effluent samples were obtained from the participants, who emptied the contents of their ileostomy pouches into Whirl-Pak (Nasco) specimen bags at predetermined time points. Immediately after each effluent collection, the sample was weighed. A small subsample was taken directly into the fixative (see Microstructural analysis), and the remaining effluent was then homogenized (HR1363 Hand Blender; Philips). For starch and sugar analysis, ∼2 g of the homogenized effluent was weighed into 4 × 15 mL BD Falcon tubes (BD Biosciences) preloaded with 95% (vol:vol) ethanol to inactivate any residual enzyme activity. These samples were stored overnight at 4°C before starch and sugar analysis. Subsamples of the homogenized effluent were also taken for determination of dry matter, and these were frozen immediately (−80°C) to prevent further degradation. Overnight samples were collected into specimen bags by the participants ad libitum and subsequently placed in absorbent padding between eutectic freezer blocks (PlusIce PCM, E-78; Phase Change Material Products) in a polystyrene box to freeze the samples at the time of collection. These frozen samples were stored for future analysis.

### Biochemical assays

Plasma glucose, serum triacylglycerol, and serum NEFA concentrations were determined on an iLab 650 auto-analyzer (Instrumentation Laboratories) with the use of Glucose Oxidase and Triglyceride IL test kits (Instrumentation Laboratories) and a Randox NEFA kit (Randox Laboratories). Serum insulin and C-peptide and plasma GLP-1, GIP, and total PYY (1–36 and 3–36 forms) concentrations were determined by standard methods as described previously (27). Cholecystokinin was determined by competitive inhibition enzyme immunoassay technique with the use of an ELISA kit (USCN Life Science).

RS was defined as the sum of starch and sugars (expressed as maltose equivalents) present in the ileal effluent, because the sugars are likely to be products of starch degradation (e.g., by microorganisms), as described in previous studies (28, 29). To determine the total amount of RS, the homogenized, ethanolic ileal effluent was centrifuged for 4 min at 12,000 × *g* at 22°C, and the resulting supernatant was analyzed for total reducing sugar, while the pellet was analyzed for total starch content. Total reducing sugar was determined by 3,5-dinitrosalicylic acid assay in which 15 μL of the supernatant (diluted in water if necessary) was transferred into a 96-well plate (Nunc, MicroWell, Fisher Scientific) to which was added 5 μL of 3M NaOH and 20 μL 3,5-dinitrosalicylic acid reagent consisting of 1.0% (wt:vol) 3,5- dinitrosalicylic acid, 1.6% (wt:vol) NaOH and 30% (wt:vol) sodium potassium tartrate, prepared in deioinized water. Standard samples containing known concentrations of maltose were also included. The plate with samples was then sealed and incubated in a water bath at 90°C for 5 min, after which 200 μL deionized water was added. The absorbance of standards and samples were then measured at 544 nm with the use of a FluoStar Optima plate reader (BMG Labtech), thereby enabling total reducing sugar concentrations to be calculated as maltose equivalents. Starch determinations were carried out with the use of a scaled-down version (18) of the Megazyme Total Starch test (Association of Official Analytical Chemists 996.11 Official Method; DMSO format) (30). Moisture content of ileal effluent was determined from the reduction in sample weight after oven-drying at 103 ± 2°C for 16 h.

Mean transit time (MTT) was calculated (28) as shown in Equation *1*, in which *t* is the time interval between ingestion and ileal recovery of RS for samples 0–*N.*


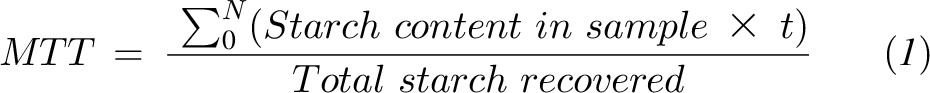


### Microstructural analysis

For examination by light microscopy, samples of ileal effluent were immersed in Karnovsky’s fixative (1.6% formaldehyde and 2% glutaraldehyde; 0.08M sodium cacodylate; pH 7.2), and left to fix at room temperature for at least 24 h. The samples were subsequently rinsed in sodium cacodylate buffer (0.1M), dehydrated through increasing concentrations of ethanol, and then infiltrated with freshly prepared LR white resin mixture (Sigma-Aldrich) prepared in 1.98% (wt:wt) benzoyl peroxide with the use of absolute ethanol as the transition solvent. Finally, resin-embedded samples were sealed in BEEM 00 capsules and polymerized at 60 ± 2°C for 24 h. The cured samples were trimmed and sectioned (0.5–1.0 μm) on an Ultracut E Reichert-Jung microtome mounted with a glass knife. Sections were stained with Lugol’s iodine [2.5% (wt:vol) I_2_ with 5% wt:vol potassium iodide] and viewed on a Zeiss Axioskop 2 mot plus light microscope. Images were captured with a Zeiss AxioCam HRc and AxioVision v3.1 microscope software (Carl-Zeiss).

### Statistical analysis and software

Power calculations were performed with G*Power v3.1.9.2 (University of Dusseldorf). Graphs and incremental AUCs were obtained with SigmaPlot 12.5 (Systat Software). Statistical analysis was performed with IBM SPSS Statistics 20.0. Data were checked for normality with the use of a Shapiro-Wilks normality test before analysis. A repeated-measures ANOVA was performed with time and meal as “within” factors to evaluate the effect of the test meals on postprandial responses. When significant meal × time effects were detected, Student’s paired *t* tests were used to identify time points with significantly different values between the 2 meals. The Bonferroni method was used to correct for multiple comparisons. All statistical analyses were performed on data obtained between 0 and 240 min, and additional subanalysis was performed for glucose, insulin, C-peptide, and GIP data obtained from AUC values calculated for the 0–120 min period, because this is the most dynamic part of the blood response curves to a carbohydrate-rich meal and is a common indicator of glycemia (1, 31). Statistically significant effects were accepted at the 95% level. Data are presented as means ± SEMs unless otherwise specified.

## RESULTS

### In vitro starch digestibility of test meals

**FIGURE 2:**
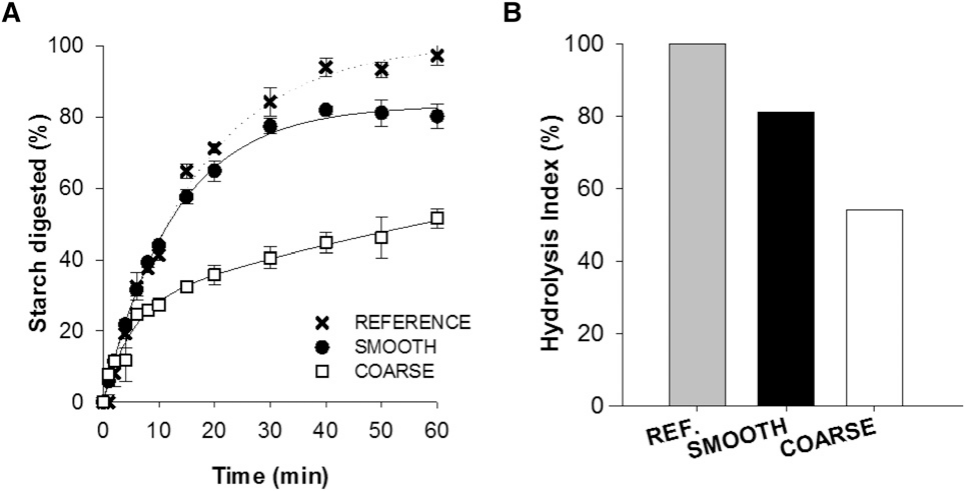
Starch digestibility (A) and HI (B) of smooth and coarse porridge endosperm relative to pure starch assessed in vitro. Digestibility curves show percentage of hydrolyzable starch digested for smooth and coarse endosperm and a highly digestible reference (i.e., pure, gelatinized durum wheat starch). In these curves, each experimental point represents the mean value from analysis performed in triplicate with vertical error bars showing SEMs. Digestibility curves of smooth and coarse porridge were significantly different (paired *t* test for incremental AUC at 60 min, *P* < 0.001). HI is the percentage of hydrolyzable starch digested at 90 min, and was calculated with the use of the previously described logarithm of slope model (18). HI values were significantly different between smooth and coarse porridge (paired *t* test, *P* = 0.03). HI, hydrolysis index; REF, reference.

The cooked particles of wheat endosperm used to make the smooth and coarse porridge meals were found to have contrasting starch amylolysis characteristics (**Figure 2**). Coarse porridge endosperm (cell wall–encapsulated starch) was digested more slowly and to a lesser extent than smooth porridge endosperm (no encapsulated starch), and both materials were less digestible than the pure starch reference (100% hydrolyzable starch). After 90 min, the amount of hydrolyzable starch digested was 33% lower for coarse porridge (HI = 54) than for smooth porridge (HI = 81) and, on this basis, a comparable difference in magnitude of the blood glucose response (incremental AUC) over 120 min was expected.

### Participant characteristics

A consort flow-chart of participants through the study is shown in **Supplemental Figure 2**. Of the 17 participants screened, 11 were randomly assigned to the present study. One participant was excluded after the first intervention because of having abnormally high fasting blood glucose concentrations (despite having a normal value at screening) and one further participant withdrew during the second intervention because of illness (abdominal discomfort). Because we could not rule out the possibility that the abdominal discomfort experienced by this participant was related to the test meal, no further interventions were attempted with this individual. A total of 9 participants (7 women and 2 men) completed the study and participant characteristics are shown in **Table 1**. Samples from all 9 participants were analyzed with regard to primary and secondary outcomes; however, some gut hormone analysis data (regarded as exploratory outcomes) were not obtained for 2 participants because of insufficient blood volumes collected and a laboratory error. Because of the repeated-measures design, gut hormone data after the 2 meals for these participants was excluded from the statistical analyses.

**TABLE 1:** Characteristics of the 9 participants (7 women and 2 men) completing the study^1^

	Value
Age, y	47.8 ± 18.0
BMI, kg/m^2^	23.9 ± 3.9
Waist:hip ratio, cm	83.3 ± 11.8:100.7 ± 6.9
Systolic/diastolic blood pressure, mm Hg	110.3 ± 16.1/70.3 ± 9.2
Plasma glucose, mmol/L	5.1 ± 0.7
Serum triacylglycerols, mmol/L	1.0 ± 0.4
Serum total cholesterol, mmol/L	4.9 ± 0.9
Dietary intake^2^	
Energy, MJ/d (kcal/d)	8.01 ± 3.26 (1938 ± 765)
Protein, % energy/d	20.4 ± 11.4
Fat, % energy/d	36.2 ± 15.1
Carbohydrate, % energy/d	40.5 ± 17.0
Dietary fiber (AOAC^3^), g/d	19.4 ± 10.9

1 Values are means ± SDs.

2 Mean values for dietary intake of nutrients and energy as analyzed by NetWisp 3.0 (Tinuviel software) were close to United Kingdom recommended guidelines (32).

3 AOAC, Association of Official Analytical Chemists.

### Postprandial blood glucose, insulin, C-peptide, and gut hormone concentrations

**FIGURE 3:**
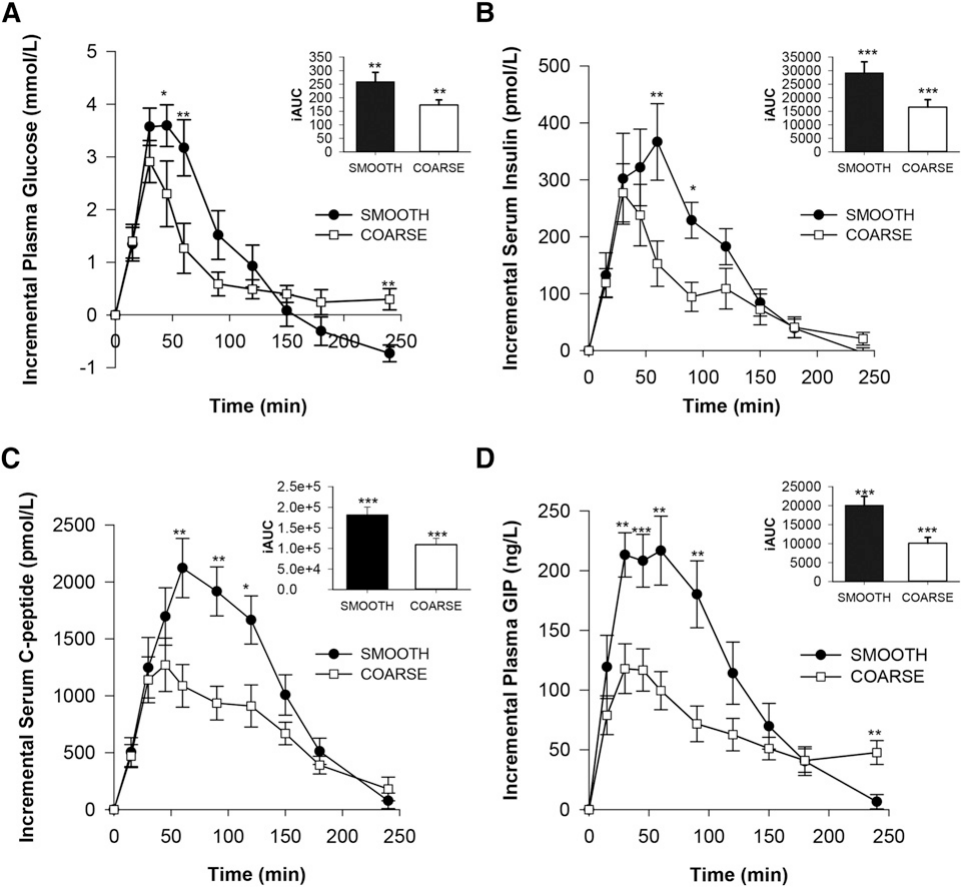
Postprandial changes in blood glucose (A), insulin (B), C-peptide (C), and GIP (D) concentrations after smooth and coarse porridge meals. Each meal provided 55.4 g of starch. Values are mean deviations from baseline ± SEMs (*n* = 9 for glucose, insulin, and C-peptide and *n* = 8 for GIP) and were analyzed by ANOVA with meal and time as factors. Meal, time, and meal × time were significant for glucose (*P* = 0.038, <0.001, and <0.001), insulin (*P* = 0.046, <0.001, and 0.035), C-peptide (*P* = 0.018, <0.001, and <0.001), and GIP (*P* = 0.024, <0.001, and <0.001) responses, respectively. Time points at which values differed significantly, *P < 0.05, **P < 0.01, and ***P < 0.001 (paired *t* test with Bonferroni corrections). Insets show the iAUC between 0 and 120 min. GIP, glucose-dependent insulinotropic polypeptide; iAUC, incremental AUC.

Plasma glucose (0–120 min), serum insulin, C-peptide, and plasma GIP concentrations were significantly lower after the coarse porridge (i.e., made with low-bioaccessibility starch) than after the smooth porridge containing high-bioaccessibility starch (**Figure 3**). A main meal effect was found over 120 min for glucose (*P* = 0.038) and over 240 min for insulin (*P* = 0.046), C-peptide (*P* = 0.018), and GIP (*P* = 0.024), but not for glucose (*P* = 0.229). Also, significant meal × time interactions over 240 min were observed for glucose (*P* < 0.001), insulin (*P* = 0.035), C-peptide (*P* < 0.001), and GIP (*P* < 0.001). After 120 min, the incremental AUC for glucose, insulin, C-peptide and GIP were 33%, 43%, 40%, and 50% lower (*P* < 0.01), respectively, after consumption of coarse porridge than after smooth porridge. The smooth porridge, which consisted of highly bioaccessible starch, elicited a rapid and large glycemic response within the first 60 min, followed by a reduction in glucose with an overshoot to nearly 1 mmol/L below the fasting value. In contrast, the coarse porridge elicited a smaller rise in blood glucose and insulin concentrations, with a much slower rate of decline after 90 min; indeed, the glucose concentrations remained above fasting even after 4 h. The peak concentrations of glucose, insulin, C-peptide, and GIP were 12%, 32%, 37%, and 60% higher (*P* < 0. 01), respectively, after smooth than after coarse porridge and were reached within 90 min of ingesting the test meals.

Although there was no main effect of the meal on GLP-1 and PYY (**Supplemental Figure 3**), significant meal × time effects were observed over 240 min for GLP-1 (*P* = 0.009) and PYY (*P* = 0.053). Plasma GLP-1 and PYY concentrations increased rapidly after test meal consumption and returned to baseline concentrations within 2 h of test meal ingestion. Peak concentrations after smooth and coarse porridge consumption were not significantly different (mean peak concentrations with 95% CIs after smooth and coarse porridge, 18.5 ± 4.8 and 17.0 ± 4.9 pmol/L for GLP-1 and 66.9 ± 10.9 and 65.0 ± 11.9 ng/L for PYY, respectively). Cholecystokinin concentrations fluctuated throughout the 4 h period, and no clear pattern was evident (data not shown).

### Postprandial serum triacylglycerol and NEFA concentrations

**FIGURE 4:**
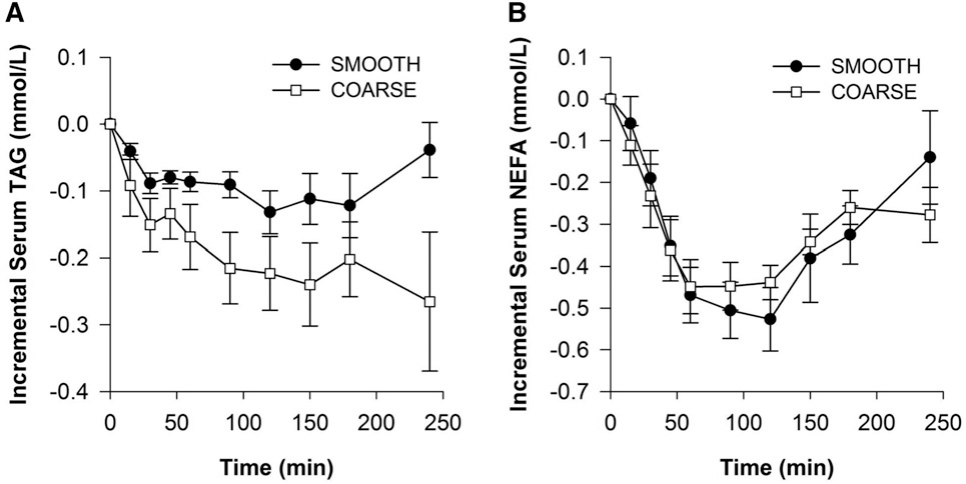
Postprandial changes in serum TAG (A) and NEFA (B) concentrations after smooth and coarse porridge meals. Each meal provided 1.56 g fat. Values are mean deviations from baseline ± SEMs (*n* = 9) and were analyzed by ANOVA with meal and time as factors. Time effects were highly significant for both TAGs (*P* = 0.004) and NEFAs (*P* < 0.001), but meal and meal × time were not significant (*P* = 0.074 and 0.100 for TAGs and *P* = 0.969 and 0.249 for NEFAs). NEFA, nonesterified fatty acid; TAG, triacylglycerol.

The pattern of triacylglycerol and NEFA responses after the test meals (1.56 g fat) are shown in **Figure 4**. After both meals, serum NEFA concentrations decreased from fasting concentration within the first 60 min, then gradually increased again toward the end of the 4-h time period. A gradual reduction in serum triacylglycerols was also observed. The effect of time on triacylglycerol and NEFA concentrations was statistically significant (*P* = 0.04 and *P* < 0.001, respectively). Statistical analysis showed that the test meals had no effect on the pattern of the response curves (meal × time effect, *P* = 0.100 for triacylglycerols and *P* = 0.249 for NEFAs); however, there was a tendency toward a lower triacylglycerol response after the coarse porridge (Figure 4A).

### Meal transit and RS in ileal effluent

The smooth and coarse porridges did not have any significant effects on the total amount of ileal output or meal transit, or on the amount of RS in ileal effluent (**Table 2**). Ileal output of RS, defined as the sum of starch and sugar recovered at the terminal ileum, followed a similar trend to dry matter over 10 h, including peaking at the same time point of 6 h (**Supplemental Figure 4**). A total of ∼3 g of RS (i.e., 5% of the starch in the test meal) was recovered at the terminal ileum during the 10 h after ingestion of both smooth and coarse porridge meals (no statistically significant differences were observed). For both test meals, the amount of RS recovered over the entire 24-h collection period, estimated from cumulative data, was higher than the 10-h values (Supplemental Figure 4). However, when considering the microstructural observations (see the next section) it seems likely that the starch measured in the overnight samples may have originated from sources other than the test meals (e.g., the evening meal). Thus, the RS recovered during the first 10 h may be more representative of the RS content of the porridge test meals than the 24-h samples.

**TABLE 2:** Outcome measures in ileal effluent up to 10 h after consumption of smooth and coarse porridge test meals^1^

	Smooth porridge	Coarse porridge
Total effluent, g/d	359 ± 255 (192, 525)	309 ± 210 (171, 446)
Moisture content of effluent, g/100g	92 ± 2 (91, 94)	91.3 ± 2 (90, 93)
Total dry matter, g/d	22 ± 7 (18, 27)	24 ± 7 (19, 28)
Total resistant starch, g/d	3.0 ± 1.1 (2.3, 3.7)	2.9 ± 1.2 (2.1, 3.7)
Starch, g/d	2.4 ± 0.9 (1.8, 3.1)	1.9 ± 0.8 (1.4, 2.5)
Sugar, g/d	0.6 ± 0.4 (0.3, 0.9)	1.0 ± 0.9 (0.4, 1.5)
Mean transit time,^2^ h	6.0 ± 0.9 (5.4, 6.6)	6.4 ± 0.6 (6.0, 6.8)

1 Values are means ± SEMs; 95% CIs in parentheses; *n* = 9. The test meal had no significant effects on the parameters listed (*P* > 0.05, paired *t* test).

2 Calculated as described in Englyst and Cummings (28).

### Microstructural observations of ileal effluent

**FIGURE 5:**
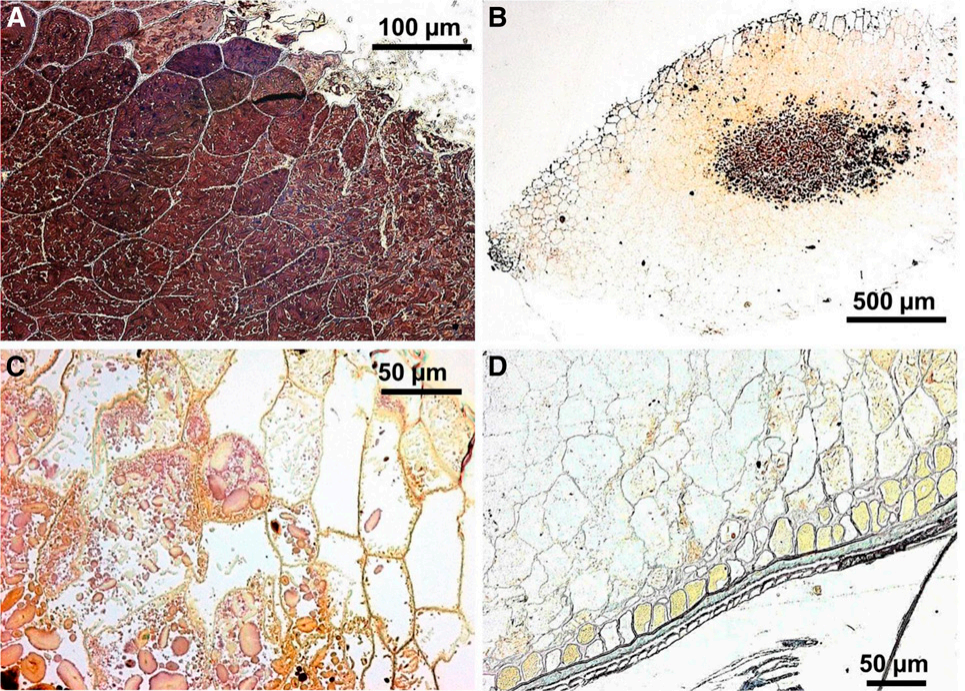
Sections of wheat endosperm particles from coarse porridge at various stages of digestion. All panels are light microscopic micrographs in which the starch was stained with 2.5% (wt:vol) Lugol’s iodine. Starch-filled wheat endosperm tissue from the cooked coarse porridge before digestion (A). Low-magnification view of a typical 2-mm wheat particle recovered in ileal effluent after 4 h (B). The staining pattern suggests a progressive digestion of starch from the particle periphery toward the core. A higher-magnification view than in panel B shows the digested edge of a particle collected from effluent after 4 h (C). Starch in the outermost cell layers (toward the right) has been digested, leaving empty cells. Typical particle remnant recovered in ileal effluent during the night (22-h gut residence time) (D). In the overnight samples, remnants of endosperm tissue were observed only when attached to adjacent outer tissue layers (aleurone, pericarp, and testa), and most of the starch had been digested.

Examples of the microstructure of wheat particles recovered from the terminal ileum after ingestion of the coarse porridge are shown in **Figure 5**. Intact particles of durum wheat of ∼2 mm diameter were evident in all samples collected throughout the day but not in the overnight samples. In many of the intact particles recovered, the starch in the outermost cell layers did not stain strongly with iodine, which indicated that the starch in these cells had been digested. Some cells containing undigested starch were present at the center of the particle and were stained a dark purple/brown with iodine (Figure 5B). Closer examination of these particles (Figure 5C) revealed that some of the peripheral endosperm cells did not appear to contain any starch, despite the presence of seemingly structurally intact cell walls. In some parts of the endosperm particles, the outer tissue layers of the original wheat grain (i.e., the pericarp, testa, and aleurone, which constitute the bran) inevitably remained after de-branning and milling, and these layers appeared largely unaffected by the digestion process (Figure 5D). Additional plant food tissues (often structurally intact) identified in the ileal effluent (images not shown) included carrots, lettuce leaves, and peas originating from foods in other meals consumed by the participants.

## DISCUSSION

The preeminence of wheat as an important staple in the human diet and its versatility as the main ingredient in a wide range of food products is well recognized (4, 33). However, some of these foods, notably wheat bread, are characterized as being high-GI products—a property that derives mainly from their starches, which are highly bioaccessible and rapidly digested (5, 8). Wheat-based products containing starch that is slowly released and digested by preservation of the structure of the cell walls (fiber) of wheat endosperm during milling could have public health benefits (e.g., improved management of type 2 diabetes).

In the current study, we investigated the effects of cell-wall encapsulation of wheat endosperm on in vitro starch bioaccessibility and digestion and on glycemia, insulinemia, and gut hormones in ileostomy participants. The nutritionally matched test meals (smooth and coarse porridge) were freshly prepared, differing only in particle size (2 mm compared with <0.2 mm), and were swallowed with minimal mastication. This design ensured that any observed metabolic effects could be attributed to the structural properties of the test meals during gastroileal digestive transit.

Our in vitro digestion experiments clearly demonstrated that starch entrapped within the cells of the coarse wheat endosperm particles was hydrolyzed (by α-amylase) at a considerably slower rate than the exposed starch in the fine flour particles. Hence, the structural integrity of dietary fiber, as intact cell walls, can strongly influence the rate at which entrapped starch is made bioaccessible and thereby affect the time course of glucose absorption into the portal blood.

On the basis of the in vitro data showing marked differences in the rate of amylolysis between coarse endosperm and flour, we hypothesized that the same materials would also elicit differences in the in vivo glycemic responses. Indeed, the coarse porridge evoked significantly lower glycemic, insulinemic, and C-peptide responses compared with the smooth porridge. The magnitude of the reductions in glycemia and insulinemia achieved in the present study were similar to, if not greater than, the glycemic reductions achieved in some previous studies with the addition of soluble fiber or use of whole grains (8, 34). Our findings therefore highlight the importance of the physical state of the dietary fiber, present as a cell wall matrix, in influencing glycemic responses (34, 35).

Microscopic examination of ileal effluent revealed that coarse (2-mm) particles of wheat endosperm retained structural integrity during the first 10 h of gastroileal transit but that the majority of encapsulated starch was eventually digested from seemingly intact cells. To our knowledge, this is the first time that such microstructural observations have been reported for wheat endosperm digested in the upper gastrointestinal tract of humans. After 10 h, the majority of the test meal had reached the terminal ileum, and only negligible amounts of RS remained (∼5% of total starch), irrespective of particle size. This is consistent with previous ileostomy studies of white wheat bread and cornflakes (28) and suggests that wheat-based food matrices, including the endosperm macroparticles used in the current study, are permeable to α-amylase during digestive transit. Notably, both smooth and coarse porridge contained the same amount of dietary fiber and RS, but the rates of starch amylolysis and subsequent glycemic responses were significantly different. We believe that the role of cell-wall encapsulation in limiting nutrient bioaccessibility provides a more specific explanation for many of the beneficial physiologic effects that have collectively been attributed to dietary fiber (12, 36).

It has been suggested that limiting the bioaccessibility of nutrients may reduce the magnitude of gut hormone responses (37, 38). The appearance of nutrients (e.g., starch digestion products) in the intestinal lumen is an important stimulus for the release of gut hormones that promote satiety (e.g., PYY and GLP-1), insulin secretion (GIP and GLP-1), and digestive secretions (e.g., cholecystokinin) (39). Some interesting trends were observed: for instance, the GIP response to the smooth porridge was 2 times that of the coarse porridge, probably reflecting the increased rate of glucose availability from duodenal starch digestion (as observed in vitro). Also, GLP-1 and PYY concentrations tended to be higher for the first 50 min after smooth porridge than after coarse porridge, but the differences were not significant. Unlike GIP and cholecystokinin, GLP-1 and PYY hormones are both secreted from L cells located predominantly in the ileum and colon, the effect of the latter being excluded in ileostomy participants. A second/sustained peak, reflecting the nutrient sensing of colonic cells, has been reported in human subjects with intact colons (40, 41). It is also noteworthy that the present study may not have been sufficiently powered to enable differences between gut hormone responses to the high- and low-bioaccessibility test meals to be demonstrated. Nevertheless, the effect of bioaccessibility on endocrine signaling is an area that has important implications for the regulation of food intake and digestion and warrants further investigation.

Although the test meals were rich in starch and contained little fat (1.6 g/portion), postprandial reductions in triacylglycerol and NEFA concentrations were observed. These reductions probably reflect the net effect of the insulin-mediated uptake of fatty acids and the inhibition of lipolysis in adipose tissue (42, 43). No significant differences were observed between the 2 meals, but, interestingly, the smooth porridge, which elicited the greatest insulin response, was associated with higher concentrations of triacylglycerols than the coarse porridge. This is paradoxical, because insulin is known to promote lipogenesis (e.g., triacylglycerol storage in adipose tissue) (44). However, it has been reported that high-carbohydrate diets can induce hypertriglyceridemia, possibly by slowing the removal of triacylglycerols (chylomicrons) from the blood (45) or by increasing hepatic VLDL release (as reported in a study of insulin-resistant subjects) (46).

Preserving the natural structural integrity (intact cells) of wheat endosperm, and indeed other edible plant tissues, during processing could provide a means of limiting the rate and/or extent of energy uptake from food postingestion and may have significant and important applications in the prevention and management of cardiometabolic diseases. Although wheat breads containing 50–80% whole grains have previously been shown to elicit lower glycemic responses than white bread (8, 19), granary bread containing such high proportions of intact grains will have a different texture and appearance from white bread and may therefore not appeal to many consumers. To our knowledge, this is the first study to demonstrate the potential to manipulate the structure of the endosperm fraction (i.e., the main component of white flour) to achieve a lower and more desirable glycemic response. Developing a low-GI “white” wheat flour could provide a new means of reducing the GI of white bread and a range of other starch-rich staple foods to positively affect public health. Further studies will be needed to better understand how other natural food structures, including *Triticum aestivum* (bread wheat), are affected by processing and digestion, but the results of the current work on durum wheat, which is used in bread- and pasta-making, demonstrate proof of principle.

There is now convincing evidence that the tissue structures of some plant foods (e.g., intact cells) can be retained to a greater or lesser extent during mastication (47) and gastroileal (10, 14, 15) and gastrocolonic (12) digestive transit and that nutrients entrapped within these structures may escape digestion and absorption to a variable extent. As a result, nutrient composition data, which does not adequately account for differences in bioaccessibility, can sometimes lead to substantial overestimations of the nutritional and caloric value of a food (48, 49). We suggest that future nutritional studies consider the structure and not just the composition of test meals.

In conclusion, these results provide the first evidence that the structural integrity of wheat endosperm is a major factor in influencing starch bioaccessibility and that manipulating the structural integrity of the endosperm has the potential to greatly influence postprandial metabolism.

## Footnotes

7 Abbreviations used: GI, glycemic index; GIP, glucose-dependent insulinotropic polypeptide; GLP-1, glucagon-like peptide 1; HI, hydrolysis index; MTT, mean transit time; NEFA, nonesterified fatty acid; PYY, polypeptide YY; RS, resistant starch.
